# Exploratory Rearing Is Governed by Hypothalamic Melanin-Concentrating Hormone Neurons According to Locus Ceruleus

**DOI:** 10.1523/JNEUROSCI.0015-24.2024

**Published:** 2024-04-04

**Authors:** Cristina Concetti, Paulius Viskaitis, Nikola Grujic, Sian N. Duss, Mattia Privitera, Johannes Bohacek, Daria Peleg-Raibstein, Denis Burdakov

**Affiliations:** Department of Health Sciences and Technology, Neuroscience Center Zürich (ZNZ), Swiss Federal Institute of Technology (ETH Zürich), Zürich 8092, Switzerland

**Keywords:** exploration, hypothalamus, innate behavior, locus ceruleus, melanin-concentrating hormone, rearing

## Abstract

Information seeking, such as standing on tiptoes to look around in humans, is observed across animals and helps survival. Its rodent analog—unsupported rearing on hind legs—was a classic model in deciphering neural signals of cognition and is of intense renewed interest in preclinical modeling of neuropsychiatric states. Neural signals and circuits controlling this dedicated decision to seek information remain largely unknown. While studying subsecond timing of spontaneous behavioral acts and activity of melanin-concentrating hormone (MCH) neurons (MNs) in behaving male and female mice, we observed large MN activity spikes that aligned to unsupported rears. Complementary causal, loss and gain of function, analyses revealed specific control of rear frequency and duration by MNs and MCHR1 receptors. Activity in a key stress center of the brain—the locus ceruleus noradrenaline cells—rapidly inhibited MNs and required functional MCH receptors for its endogenous modulation of rearing. By defining a neural module that both tracks and controls rearing, these findings may facilitate further insights into biology of information seeking.

## Significance Statement

Information seeking is a fundamental behavior related to cognition and neuropsychiatric states. The neural circuits underlying it are still unclear. We show that hypothalamic neurons that make melanin-concentrating hormone neurons (MNs) are active during a well-known rodent analog of information seeking—rearing on hind limbs—and contribute to driving these acts of rearing. We also find that locus ceruleus noradrenergic neurons, known for mediating stress responses, inhibit MNs, thus linking stress and information seeking. These results identify a neural substrate of information seeking and provide insights into how the brain toggles between priorities.

## Introduction

Animals are preprogrammed to perform certain self-paced actions that can be viewed as dedicated efforts to scan their environment for information. Examples include lifting your head up or standing on tiptoes to look around in humans or exploratory unsupported rearing on hind legs in quadrupeds such as rodents (Sutherland et al., 1982, 1983; Eilam and Golani, 1989; Dielenberg and McGregor, 2001; Lever et al., 2006; Mun et al., 2015; Sturman et al., 2018). This type of information seeking, which often does not have an immediate object or goal, is fundamental for psychology and evolution, since it provides survival advantages by improving cognitive models of the environment, enabling optimal solutions to future problems in naturalistic circumstances (Eilam and Golani, 1989; Lever et al., 2006; Peterson and Verstynen, 2022). Studies of information-seeking behaviors and their neural underpinnings are thus seen as critical for mechanistic understanding of biological intelligence (Lever et al., 2006; Mun et al., 2015; Sturman et al., 2018; Layfield et al., 2023). Unsupported rearing in rodents has been an important model for such studies, providing fundamental insights into neural bases of cognition (Crusio et al., 1989; Crusio, 2001; Lever et al., 2006; Mun et al., 2015; Layfield et al., 2023). Importantly, these insights are considered relevant to humans, since these spontaneous acts of exploratory behavior (henceforth, simply rearing or rears) in rodents are considered analogous to human acts of curiosity and exploration (Dielenberg and McGregor, 2001; Lever et al., 2006; Kang et al., 2008; Mun et al., 2015; Layfield et al., 2023). Furthermore, rearing is readily and widely observed in the most frequently used test in rodent behavior labs around the world, the open-field test. A detailed understanding of behaviors displayed by rodents in this test is a prime focus of the current revolution in state-of-the-art approaches to artificial intelligence-assisted ethology (Berman et al., 2014; Wiltschko et al., 2015, 2020; Hsu and Yttri, 2021; Bordes et al., 2023; von Ziegler et al., 2023). There is now a renewed interest in mouse rearing, with recent descriptions of this behavior and its sensitivity to stress attracting a lot of attention due to the potential use of rearing quantification as a valuable metric for high-throughput preclinical studies of neuropsychiatric states (Sturman et al., 2018; Chen et al., 2023; Shan et al., 2023).

Despite these notable past, present and likely future impacts of studying rodent rearing behavior on basic and translational neuroscience, neural signals, and circuits controlling rearing remain largely unknown. Classic and recent studies suggest recruitment of hippocampal activity during rearing (Crusio et al., 1989; Harley and Martin, 1999; Crusio, 2001; Deacon et al., 2002; Barth et al., 2018; Layfield et al., 2023; Privitera et al., 2024), in line with traditional conceptualization of this region as a center for spatial exploration (O’Keefe and Nadel, 1979). Recently, spatial exploration in rodents was also found to involve phasic activation of brain regions not traditionally implicated in this process, such as the lateral hypothalamus (LH; González et al., 2016a; Blanco-Centurion et al., 2019; Kosse and Burdakov, 2019). However, it has not been tested whether these hypothalamic upstates correspond to rears versus other behaviors (such as running, licking, and grooming) that are closely interspersed in self-paced behavioral sequences.

Here, while investigating the fine timing of specific behaviors in relation to LH activation, we came upon profound activation of melanin-concentrating hormone (MCH) cells during rearing. MCH cells (MNs) are only found in the LH, the neighboring incerto-hypothalamic area, and the dorsolateral part of the zona incerta (Bittencourt et al., 1992; Sita et al., 2007; Bittencourt, 2011, 2022; Diniz et al., 2019; Diniz and Bittencourt, 2019). However, they project their axons brain-wide, releasing the MCH neuromodulator peptide that acts on dedicated G-protein-coupled MCH receptor (only MCHR1 in the mouse) that exerts powerful effects on synaptic plasticity (Monzon et al., 1999; Varas et al., 2002, 2003; Adamantidis et al., 2005; Adamantidis and Lecea, 2009; Pachoud et al., 2010; Barillier et al., 2015; Izawa et al., 2019; Oh et al., 2019; Burdakov and Peleg-Raibstein, 2020; Harris et al., 2022; Liu et al., 2022). The MCH system is also present and considered important in humans (Mouri et al., 1993; Blouin et al., 2013; Kiss et al., 2018; Vawter et al., 2019; Mladinov et al., 2021; Barbosa et al., 2023). Due to this importance of MNs and of understanding rearing, we performed further causal experiments, including optogenetic experiments, manipulating MCH neurons and their putative modulator, the stress-implicated locus ceruleus (LC; van den Pol et al., 2004), and pharmacological experiments aimed at suppressing endogenous MCHR1 activation. Below, we present and discuss the correlative and causal links between MCH system and rearing that emerged from these experiments.

## Materials and Methods

### Animal experimentation

All animal procedures were performed in accordance with the Animal Welfare Ordinance (TSchV 455.1) of the Swiss Federal Food Safety and Veterinary Office and were approved by the Zurich Cantonal Veterinary Office. Mice were kept on a standard chow and water *ad libitum* and on a reversed 12 h light/dark cycle. Experiments were performed during the dark phase, and time of day was counterbalanced between groups. Adult males and females (at least 8 weeks old) in comparable proportions were used in each group. No differences in rearing modulation and rearing-associated MCH activity were observed between genders, which were therefore aggregated in the analyses.

### Viral vectors and histology

The specific targeting of the GcaMP6s calcium sensor and opsins to MNs and LC noradrenergic neurons was performed using genetic tools described and histologically validated in previous studies (Carter et al., 2010; Kosse and Burdakov, 2019).

To target GcaMP6s to MNs, we injected an AAV vector carrying the 0.9 kb preproMCH gene promoter AAV9.pMCH.GcaMP6s.hGH [1.78 × 10^14^ gc/ml; Vigene Biosciences, characterized to target MCH cells with >90% specificity in Kosse and Burdakov (2019)] into the LH of C57BL6 mice. Over 90% of MCH cells expressed GCaMP [549/602 cells from three brains, validated using histological protocol described in Kosse and Burdakov (2019)].

To target the excitatory opsin ChrimsonR to MNs, we injected AAV-EF1a-DIO-ChrimsonR-mRuby2-KV2.1-WPRE-SV40 (5 × 10^11^ gc/ml; Addgene) bilaterally into the LH of the previously characterized and validated MCH-Cre mice (Kong et al., 2010), which were bred in het-WT pairs with C57BL/6 mice. Confirmation of ChrimsonR expression was performed by histology for the colocalization of mRuby and MCH staining as described previously (Kosse and Burdakov, 2019; Fig. 3*A*; >75% of MCH cells expressed ChrimsonR, 123/161 cells from three brains).

To target excitatory and inhibitory opsins to LC noradrenergic neurons, we injected the Cre-dependent constructs AAV-EF1a-DIO-ChrimsonR-mRuby2-KV2.1-WPRE-SV40 (5 × 10^11^ gc/ml; Addgene) and AAV-EF1a-DIO-eNpHR3.0-mCherry-WPRE (5 × 10^12^ gc/ml; UNC Vector Core) unilaterally in the LC of C57BL/6-Tg(Dbh-icre)1Gsc (MGI ID: 4355551), expressing Cre recombinase in LC noradrenergic neurons, as characterized in previous studies (Stanke et al., 2006; Parlato et al., 2007).

For each opsin-expressing cohort of mice, corresponding control mice were littermates who underwent the same surgery, without opsin AAV injection. For every experiment, mice in each treatment group and corresponding control group were subjected to the same behavioral experimentation on the same day with a counterbalanced design. In fiber photometry and optogenetic experiments, fiber tip placements and viral expression were verified by postrecording examination of brain slices, and mice with misplaced fibers or those that lacked expression were excluded from analysis (typically this was <1% of animals).

### Stereotaxic surgery

For stereotaxic brain injections, mice were anesthetized with isoflurane and injected with Metacam (5 mg/kg of body weight, s.c.) for analgesia. In a stereotaxic frame (Kopf Instruments), a craniotomy was performed, and a 33-gauge needle mounted on a Hamilton syringe was used to inject AAV vectors.

To target the LH, an injection (150 nl at a rate of 50 nl/min) was administered in one or both hemispheres (bregma, AP −1.35 mm; ML ±0.90 mm; DV 5.30 mm; 0° angle—or bregma, AP −1.35 mm; ML ±1.90 mm; DV 5.30 mm; 10° angle), and fiber-optic implants were placed above the injection site (bregma, AP −1.35 mm; ML, ±0.90 mm; DV, 5.00 mm; 0° angle—or bregma, AP −1.35 mm; ML, ±1.90 mm; DV, 5.10 mm; 10° angle) based on González et al. (2016a,b) and Kosse and Burdakov (2019) (locations of fiber tips were confirmed by histology and found to be within 200 μm of stated coordinates in the mice included in this study). To target the LC, two injections (300 nl at a rate of 50 nl/min) were administered in one hemisphere (bregma, AP −5.3 mm for females, −5.4 mm for males; ML ±0.90 mm; DV 3.7 mm, 3.4 mm), and a fiber-optic implant was placed above the injection site (bregma, AP −5.3 mm for females, −5.4 mm for males; ML ±0.90 mm; DV 3.3 mm) based on Zerbi et al. (2019) and Privitera et al. (2024). Whenever unilateral targeting was used, hemispheres were counterbalanced among animals. For inclusion of animals in experiments, the fiber tip had to be within a 0.25 mm radius of the intended target, and there had to be evident viral expression. For experiments involving bilateral infusions and implants, these criteria had to be met in both hemispheres. Based on these criteria, no animals had to be excluded.

Before the experiments, mice were allowed to recover from surgery for at least 10 d.

### Fiber photometry

Fiber photometry was performed using the Doric fiber photometry system, in lock-in mode using simultaneous illumination with two LEDs (405 and 465 nm excitation, oscillating at 334 and 471 Hz, respectively; average power, ∼100 μW at the fiber tip). Fluorescence produced by 405 nm excitation provided a real-time control for motion artifacts (Kim et al., 2016).

### Optogenetics

The excitatory opsin ChrimsonR was activated by a red laser [635 nm; Laserglow Technologies; 30 Hz, 10 ms ON, based on Jego et al. (2013) and Blanco-Centurion et al. (2016)] and the inhibitory opsin eNpHR was activated by a yellow laser (589 nm; Laserglow Technologies; continuous wave), both yielding ∼7 mW light power at the fiber tip. The illumination protocol for ChrimsonR was 3 min laser OFF, 3 min laser ON, 3 min laser OFF, based on McCall et al. (2015), and carried out once. Since MN activity recovers after ∼60–100 s (Figs. 2–4*C*), behavioral data were analyzed in the first 60 s of ChrimsonR stimulation (Fig. 6*D–F*), and the baseline behavior before laser illumination was analyzed in the 60 s before that (Fig. 5*E*). The illumination protocol for eNpHR was 1 min ON followed by 1.5 min OFF × 6 times based on Mattis et al. (2012) and Wiegert et al. (2017), with a ramping down offset over 100 ms to avoid rebound excitation, based on Mahn et al. (2016).

### Pharmacology

The MCH receptor antagonist SNAP-94847 hydrochloride (Tocris Bioscience, 3347) was administered intraperitoneally at a dose of 20 mg/kg, dissolved in distilled water with 10% DMSO (99.5%, PanReac AppliChem, 131954.1611) and 30 mg/ml of β-cyclodextrin (Sigma, H107), based on Marsteller et al. (2009). Distilled water with 10% DMSO and 30 mg/ml of β-cyclodextrin was used as vehicle. Intraperitoneal administration of SNAP or vehicle was done 45 min prior to behavioral testing, as in Kosse and Burdakov (2019). Mice were habituated 2× to intraperitoneal injections prior to experimentation.

### Open field

Open-field experiments were carried out in a 35 × 35 × 35 cm gray plexiglas box, under a ∼40 lux illumination, to ensure a nonthreatening environment favorable to the display of rearing behavior in mice (Mun et al., 2015). Video was recorded using a camera (Basler acA1300-200um, Chromos Industrial) for a duration of 20 min. In Figure 5*I–N*, data were analyzed in the time interval 3–6 min after last laser OFF. In all cases, two sessions were carried out for each mouse and for each condition and then averaged (except for Fig. 2, where each mouse received each treatment once, and one vehicle mouse was excluded because of a technical error in intraperitoneal injection, and for Fig. 5*I–N*, where one session was carried out). Mice were habituated to the apparatus before testing.

### Licking

Licking behavior was recorded in a separate chamber to avoid potential interference between self-paced behaviors in the neutral environment of the open-field and food-driven motivation. Mice were deprived of food during the previous light phase and then given access to food for 2 h before testing, to ensure a consistent food intake. The test was carried out in a 19 × 19 × 35 cm plexiglas chamber installed in a ventilated, sound-insulated chest (Coulbourn Instruments) and equipped with an infrared camera (Basler acA1300-200um, Chromos Industrial), a metal spout connected to a peristaltic pump (WPM1, PeriPump) driven by an Arduino board (Arduino UNO), and a capacitive touch sensor for the detection of licking (AT42QT1011, SparkFun Electronics). Fifteen microliters of liquid food (strawberry milkshake) were delivered in 1 min intervals for a total of 20 times. The output of the touch sensor was used to identify licking bouts. Each bout was defined as a cluster of consecutive licks following delivery of liquid food; bouts starting before the liquid food was made available were excluded from analysis. Mice were habituated separately to the chamber and to the milkshake in their home cage before testing.

### Fear conditioning

The test was carried out in an operant chamber (model E10-10; Coulbourn Instruments) installed in a ventilated, sound-insulated chest and equipped with a grid floor made of stainless steel rods (4 mm diameter). Scrambled electric shocks with a 0.5 mA intensity were delivered through the grid floor (model E13-14; Coulbourn Instruments). A tone (2.9 kHz, 90 dB, 30 s) was delivered through intrachamber speakers. The chamber had a total floor area of 30 cm × 25 cm and a height of 29 cm, but the mouse was confined to a rectangular 17.5 cm × 13 cm region in the center, defined by a clear plexiglas enclosure. The tone was immediately followed by a 2 s footshock and an ITI of 90 s for a total of seven pairings. This protocol was initially executed and validated using eight WT mice. Upon exposure to the same tone in an open field, these mice demonstrated substantial freezing behavior in response to this stimulus within that context, thereby confirming its effectiveness as an acute stressor (Extended Data Fig. 5-3C). The same procedure was thus used on the LC optoinhibition group and their controls.

### Video tracking and classification of behaviors in the open field

Specific behaviors were identified using a classifier based on a convolutional neural network (CNN) as in Viskaitis et al. (2022). Briefly, the CNN was trained using >4,000 movement images, generated as an RGB combining the current frame, 10 frames prior and 10 frames after (video frame rate was 30 fps), and labeled by the experimenter. The labeled frames were split between a training dataset and a validation dataset, and the training was considered complete when reaching an accuracy of ∼90% on the validation dataset, which was not used in training. The trained network was then used to classify whole experiment videos (with a sampling rate of 3 Hz). Because this tool generates motion-based RGB images, we could identify both static and dynamic behaviors. Behaviors were defined as follows: locomotion, whole body moving forward, all paws on the ground; turning, whole body rotating around the center point, all paws on the ground; immobility, body not moving, all paws on the ground, head level to the floor; grooming, body appears round and curled up, head moving, ears facing downward; and rearing, front paws are lifted, body appears shortened, head is facing up and ears backward, no vertical surface supports the body. Supported rearing was not quantified, throughout the manuscript unsupported rearing is referred to as “rearing” for simplicity. Behavioral events were defined as uninterrupted instances of a displayed behavior. Behavioral events with a duration <1 s were excluded from analysis.

### Pupillometry

To confirm expression of the excitatory opsin ChrimsonR in LC noradrenergic neurons, we performed a pupillometry test (Fig. 5*B*) as in our previous work (Grujic et al., 2022). Briefly, animals were anesthetized using 2% isoflurane, their pupil was recorded with an infrared camera (20 fps), and pupil size was analyzed using DeepLabCut. Optogenetic stimulation was delivered in trains of 20 Hz and 30 s every 2–2.5 min.

### Experimental design, data analysis, and statistics

Experimental design can be found under each section of the Materials and Methods relative to behavioral testing, and their rationale can be found in the Results section. Sample sizes are specified in figure legends. Statistical tests and descriptive statistics were performed as specified in Results and the figure legends.

**Figure 1.**
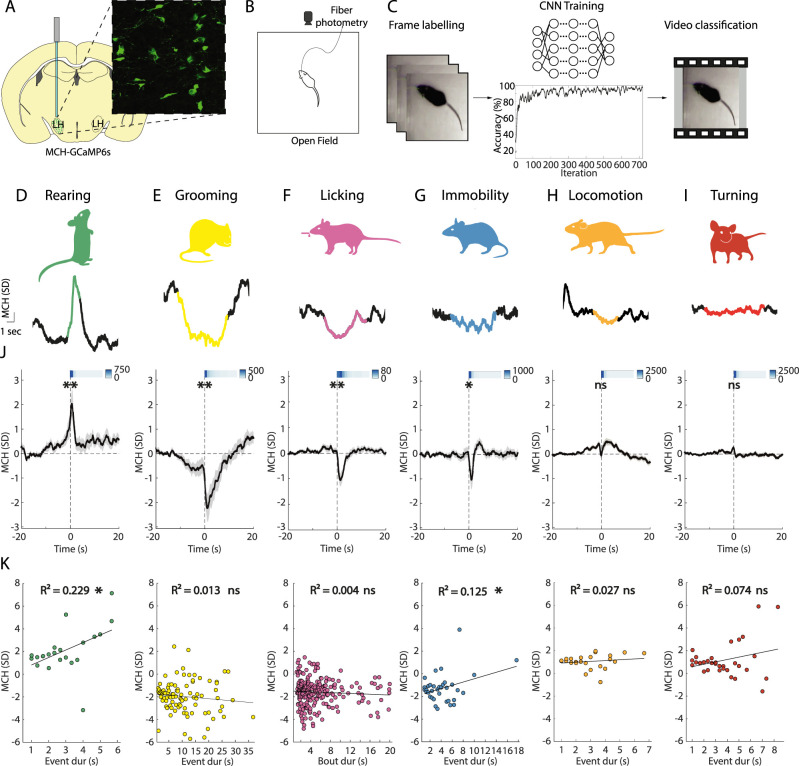
MN activation patterns aligned to initiation of self-paced behaviors. ***A***, Targeting scheme and expression of GCaMP6s in MNs. ***B***, Schematic of the open-field experimental paradigm, with video tracking and fiber photometry recording. ***C***, Workflow for behavioral classification using a CNN (see Results and Materials and Methods for details). ***D–I***, Examples of MN activity simultaneous to various self-paced behaviors. ***J***, ***K***, Behavior-associated MN activity as recorded with fiber photometry in the open field. ***J***, Each plot is an average across recording sites and behavioral events. Rearing **p = 0.0017, grooming **p = 0.0013, licking **p = 0.0011, immobility *p = 0.0367, locomotion ns *p* = 0.0927, turning ns *p* = 0.4076; one-sample *t* tests; *n* = 26 recording sites from 16 mice. The bars on the top right of each graph are heatmaps representing the temporal distribution of behavioral events (heatmap units are raw numbers of events). Data are presented as mean ± SEM. ***K***, Correlation between the amplitude of MN activity and event duration of the corresponding behavior. Rearing *p = 0.024, *R*^2^ = 0.229; grooming ns *p* = 0.275, *R*^2^ = 0.013; licking ns *p* = 0.306, *R*^2^ = 0.004; immobility *p = 0.038, *R*^2^ = 0.125; locomotion ns *p* = 0.442, *R*^2^ = 0.027; turning ns *p* = 0.125, *R*^2^ = 0.074. The black line represents linear regression. ns, *p* > 0.05; *p < 0.05; **p < 0.01.

In fiber photometry experiments, to produce the plotted % Δ*F*/*F* values, the raw 405 nm excited signal was fitted to the 465 nm excited signal, and then the % Δ*F*/*F* time series was calculated for each session as [100 * (465 signal − fitted 405 signal)/fitted 405 signal], based on Lerner et al. (2015). Data were *z*-scored to its baseline, based on Karnani et al. (2020; the baseline interval being −20 to −10 s for fiber photometry data aligned with self-paced behaviors in Fig. 1 and −50 to 0 s for fiber photometry data aligned with laser onset in Fig. 5; *t* = 0 s indicates the start of the behavior).

In Figure 1*J–K*, the amplitude was calculated as mean activity in the interval 0–1 s. In Figure 1*K*, using Bonferroni’s correction for multiple testing, *p* < 0.025 was considered significant. In Figure 1*L* each data point was obtained by calculating the maximum or minimum value in the time interval 0–1 s for each behavioral event (using positive amplitude for behaviors associated with a positive or no deflection and negative amplitude for those associated with a negative deflection, based on the results in Fig. 1*J*) and averaging amplitudes for each duration bin of the behavior for each mouse. The values of *p* and *R*^2^ were calculated using Pearson's correlation.

For behavioral data analysis, for each mouse, percent time was calculated using the formula [100 * sum (Event Duration))/Total Time; event frequency was calculated as count (Event Start)/Total Time; event duration was calculated as average (Event Duration)].

Data are presented as mean ± SEM, and a *p* value <0.05 was considered to indicate significance. Statistical significance was assessed using one-sample or two-sample *t* test, as specified in figure legends. All *t* tests were two-tailed, except for Figure 5*D–F*,*I–N*, where previous results (Fig. 5*C*,*H*, respectively, together with Figs. 3, 5) led to the formulation of directional hypotheses and the use of one-tailed *t* tests. All data processing and analysis were performed using custom scripts written in MATLAB R2022b (MathWorks).

### Code accessibility

Custom codes used for data analysis are available from the corresponding author on reasonable request.

## Results

### MNs report rearing behavior

To investigate the natural activity of MNs during various self-paced behaviors, we performed fiber photometry using the calcium indicator GCaMP6s under the *Pmch* promoter (Fig. 1*A*) while video tracking mouse behavior in an open field (Fig. 1*B*). We used a machine learning behavioral classifier tool (Viskaitis et al., 2022) based on a CNN to identify self-paced behavior (Fig. 1*C*). We defined five fundamental self-paced behaviors based on specific criteria (see Materials and Methods): rearing, grooming, immobility, locomotion, and turning. The output of the classifier was then used to identify neuronal activity simultaneous with self-paced behavioral events. An additional behavior, licking, was recorded in a separate chamber equipped with a capacitive touch sensor to detect licking from a spout through which liquid food was delivered. Figure 1, *D–I*, shows examples of MN activity corresponding to the behaviors. Using this approach, we were able to analyze behavior-associated neuronal activity with high accuracy and temporal resolution (∼90% and 3 Hz).

MN activity showed different profiles across the behavioral variables (Fig. 1*J*). MN activity significantly increased during rearing behavior; significantly decreased during grooming, licking, and immobility; but was unchanged during locomotion and turning. We also analyzed whether there is correlation between the amplitude of MN activity and event duration of the corresponding behavior (Fig. 1*K*). The data showed a significant correlation between the duration of rears and the amplitude of rearing-associated MCH activity. The association between the immobility-associated MCH signal reduction and immobility event duration was also significant, but weaker. Together, these data show that, during spontaneous behavioral sequences, MN activity is increased during self-paced rears, and rearing-associated MCH amplitude positively correlates with the duration of rears.

### MNs control rearing behavior

**Figure 2.**
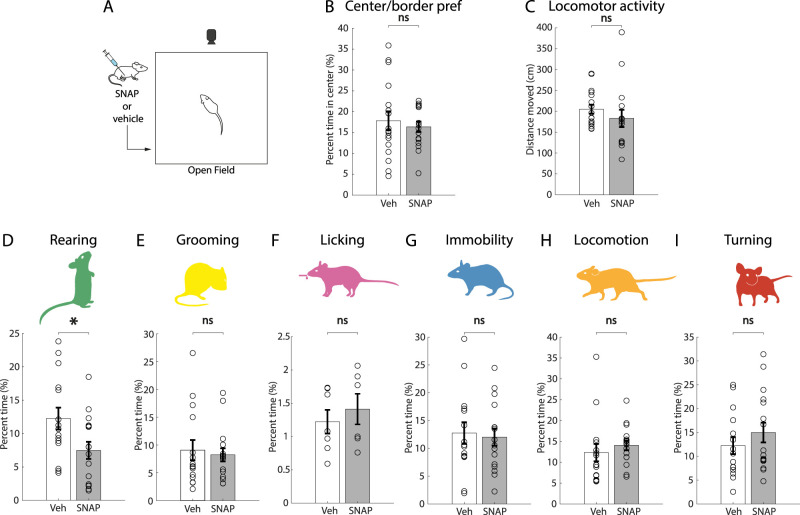
Effects of MCHR1 antagonist SNAP on self-paced behaviors. ***A***, Schematic representation of the experimental paradigm. ***B***, ***C***, Effect of treatment with SNAP (20 mg/kg) versus vehicle on center/border preference (ns *p* = 0.5903), an indicator of anxiety-like behavior, and locomotor activity in the open field (*p* = 0.3386; unpaired *t* test; *n* = 15 vehicle mice, 16 SNAP mice). ***D–I***, Effect of treatment with SNAP (20 mg/kg) versus vehicle on time spent performing each behavior. Rearing *p = 0.0223; grooming ns *p* = 0.9249; licking ns *p* = 0.5211; immobility ns *p* = 0.9110; locomotion ns *p* = 0.4626; turning ns *p* = 0.4574; unpaired *t* test, *n* = 15 vehicle mice, 16 SNAP mice for all behaviors except licking where *n* = 7 vehicle mice and 6 SNAP mice. Data are shown as mean ± SEM. ns, *p* > 0.05; *p < 0.05; **p < 0.01.

To investigate whether and how the endogenous MCH activity influences the tested behaviors, we administered an antagonist of MCHR1 (the only MCH receptor in mice; Forray, 2003), SNAP-94847 (20 mg/kg) or vehicle, via intraperitoneal injection before testing (Fig. 2*A*). SNAP-94847 did not affect center–border preference (a measure of anxiety-like behavior) or locomotor activity (Fig. 2*B*,*C*). However, mice treated with SNAP-94847 showed a significant decrease in time spent rearing, compared with vehicle-injected mice (Fig. 2*D*). No other behavior was affected by treatment with the MCHR1 antagonist (Fig. 2*E–I*). These data suggest that the MCH receptor is involved in promoting rearing behavior and that this effect is not due to potential locomotion or anxiety-related effects of SNAP.

**Figure 3.**
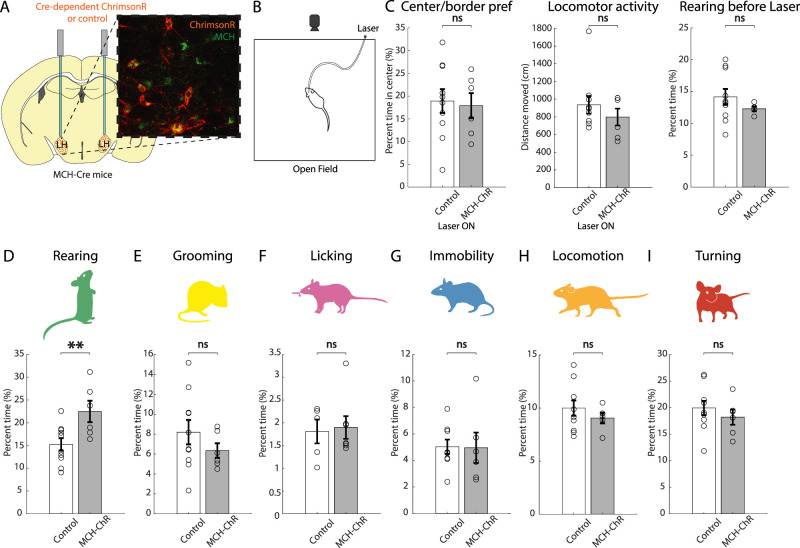
Effect of optostimulation of MNs on self-paced behaviors. ***A***, Targeting scheme and expression of the excitatory opsin ChrimsonR in MNs. ***B***, Schematic representation of the experimental paradigm (stimulation 635 nm, 30 Hz, 10 ms ON, 7 mW, 3 min OFF–3 min ON–3 min OFF). ***C***, Effect of laser light stimulation in ChrimsonR-expressing mice versus control mice on center–border preference (ns *p* = 0.8019), locomotor activity (ns *p* = 0.3692) during laser stimulation, and rearing levels before laser stimulation (ns *p* = 0.8116; unpaired *t* test). ***D–I***, Effect of laser light stimulation in ChrimsonR-expressing mice versus control mice on time spent performing each behavior. Rearing **p = 0.0062; grooming ns *p* = 0.8521; licking ns *p* = 0.2286; turning ns *p* = 0.7925; locomotion ns *p* = 0.7965, immobility ns *p* = 0.5262; unpaired *t* test; *n* = 5 ChrimsonR-expressing mice and 10 control mice, except for licking where *n* = 7 ChrimsonR-expressing mice and 5 control mice. Data are shown as mean ± SEM. ns, *p* > 0.05; *p < 0.05; **p < 0.01.

In view of these data, and since MNs are thought to be the only source of MCH in the brain (Broberger et al., 1998), we hypothesized that MN activation may selectively increase rearing behavior. To test this, we injected a Cre-dependent excitatory opsin, ChrimsonR, in the LH of MCH-Cre^+^ mice (Kong et al., 2010; Fig. 3*A*). We then recorded self-paced behaviors while delivering bilateral laser light to the LH of MCH-ChrimsonR-expressing and control mice (Fig. 3*B*). Optogenetic activation of MNs did not affect anxiety-like behavior or locomotor activity (Fig. 3*C*,*D*). Rearing levels before optogenetic stimulation were not different between groups (Fig. 3*E*). Rearing was significantly increased by optogenetic stimulation of MNs compared with controls (Fig. 3*D*), but no other behavior was affected (Fig. 3*E–I*).

**Figure 4.**
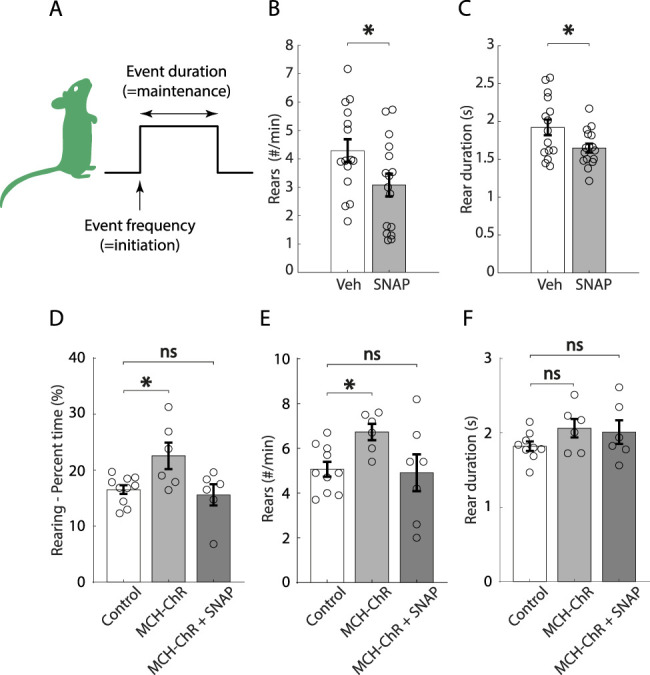
Further dissection of effects of MCH system manipulations on rearing behavior. ***A***, Graphical illustration of behavioral microstructure studied. ***B***, ***C***, Effect of treatment with SNAP (20 mg/kg) versus vehicle on rear frequency (*p = 0.0397) and duration (*p = 0.0279; unpaired *t* test, *n* = 15 vehicle mice and 16 SNAP mice). ***D–F***, Effect of laser light stimulation in ChrimsonR-expressing mice, in the absence or presence of SNAP (20 mg/kg), versus control mice, on rearing time (one-way ANOVA, *F* = 5.211, *p* = 0.0157, Dunnett's post hoc test, control vs MCH-ChR *p* = 0.0217, control vs MCH-ChR + SNAP *p* = 0.8797), rear frequency (Welch one-way ANOVA, *W* = 4.359, *p* = 0.0386, Dunnett's post hoc test, control vs MCH-ChR *p = 0.0187, control vs MCH-ChR + SNAP ns *p* = 0.6382), and duration (Welch's one-way ANOVA, *W* = 3.388, *p* = 0.0759, Dunnett's post hoc test, control vs MCH-ChR ns *p* = 0.1699, control vs MCH-ChR + SNAP ns *p* = 0.1065; *n* = 6 ChrimsonR-expressing mice and 10 control mice; stimulation 635 nm, 30 Hz, 10 ms ON, 7 mW, 3 min OFF–3 min ON–3 min OFF). Data are shown as mean ± SEM. ns, *p* > 0.05; *p < 0.05; **p < 0.01.

The time spent performing a given behavior is a result of behavioral event frequency (a measure of behavior initiation) and duration (a measure of behavior maintenance). To gain more understanding into how the MCH system regulates rearing, we therefore examined these finer elements of rearing temporal microstructure (Fig. 4*A*). We found that both frequency and duration of rears are decreased by SNAP compared with vehicle treatment (Fig. 4*B*,*C*). In turn, optogenetic activation of MNs results in a significant increase in frequency but not duration (Fig. 4*E*,*F*). Finally, given that MNs are known to express several neurotransmitters in addition to the neuromodulator MCH (Jego et al., 2013; Chee et al., 2015; Schneeberger et al., 2018), we asked whether the rearing-increasing effects of MCH cell optostimulation require MCH receptors. We found that SNAP prevented MCH cell optostimulation from increasing rearing behavior time and frequency (Fig. 4*D*,*E*). The fact that treatment with SNAP reduced rearing duration but optogenetic activation of MCH neurons did not increase duration suggests that chronic MCH receptor tone is necessary to maintain normal duration of rearing behavior, but acute optogenetic activation was not sufficient to increase this behavioral metric. Taken together, these results suggest that the ability of MNs to control rearing requires MCH receptors; the MNs and MCH receptors are specific modulators of rearing behavior.

### The MCH system as an effector of noradrenergic influence on rearing

**Figure 5.**
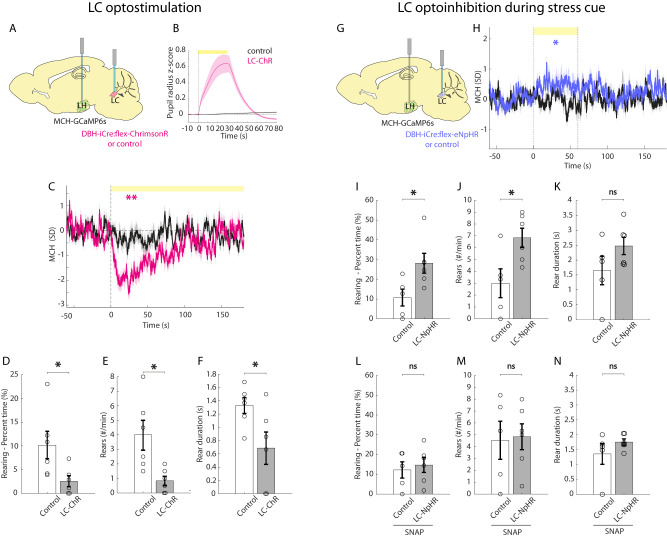
Effects of bidirectional optogenetic manipulations of LC noradrenergic neurons on MNs and rearing. ***A***, Schematic for targeting GCaMP to MNs and ChrimsonR to LC noradrenaline cells. ***B***, Pupil diameter response to laser illumination of LC in LC-ChrimsonR and control mice. ***C***, Fiber photometry response of MNs to laser illumination of the LC in LC-ChrimsonR mice (**p = 0.001, magenta) and control mice (ns *p* = 0.6247, black; one-sample *t* tests on baseline-subtracted average activity during the first 60 s of laser illumination; *n* = 12 recording sites from 6 mice for LC-ChrimsonR, *n* = 12 recording sites from 6 mice for controls; stimulation 635 nm, 30 Hz, 10 ms ON, 7 mW, 3 min OFF–3 min ON–3 min OFF). ***D–F***, Rearing behavior during MN activity inhibition caused by optostimulation of LC noradrenergic neurons in LC-ChrimsonR mice compared with that in control mice. Rearing percent time *p = 0.0182; rear frequency *p = 0.0071; rear duration *p = 0.0201; unpaired *t* test; *n* = 6 LC-ChrimsonR expressing mice and 6 control mice; stimulation 635 nm, 30 Hz, 10 ms ON, 7 mW, 3 min OFF–3 min ON–3 min OFF. ***G***, Targeting schematic of MCH-dependent GCaMP in the LH and Cre-dependent inhibitory opsin eNpHR in the LC of DBH-iCre mice. ***H***, Fiber photometry response to laser illumination of the LC and simultaneous stress cue in LC-eNphR mice (*p = 0.0484) and control mice (ns *p* = 0.7465; one-sample *t* tests; *n* = 6 eNpHR-expressing mice and 5 control mice; stimulation 589 nm, CW, 7 mW, 1 min ON followed by 1.5 min OFF x 6 times). ***I–K***, Rearing behavior following laser illumination of the LC and simultaneous stress cue in LC-eNpHR and control mice. Rearing percent time *p = 0.0151; rear frequency *p = 0.0121; rear duration ns *p* = 0.0796, *n* = 6 eNpHR-expressing mice and 5 control mice. ***L–N***, Rearing behavior in SNAP-treated mice following acute stress and laser illumination of the LC in LC-eNpHR and control mice. Rearing percent time ns *p* = 0.3343; rear frequency ns *p* = 0.4386; rear duration ns *p* = 0.1328; unpaired *t* test; *n* = 6 eNpHR-expressing mice and 5 control mice; stimulation 589 nm, CW, 7 mW, 1 min ON followed by 1.5 min OFF × 6 times. Data are shown as mean ± SEM. ns, *p* > 0.05; *p < 0.05; **p < 0.01. Further data are provided in Extended Data Figures 5-1, 5-2, and 5-3.

Stressful/threatening environments can suppress rearing (Lever et al., 2006; Mun et al., 2015; Sturman et al., 2018). Under stressful circumstances, LC noradrenergic neurons are thought to mediate central and peripheral responses to stress (Valentino and Bockstaele, 2008; Benarroch, 2018; Morris et al., 2020; Poe et al., 2020), and LC activation in the open field can suppress rearing (Privitera et al., 2024). Therefore, we sought to investigate whether LC noradrenergic neurons inhibit MNs in vivo and hypothesized that optogenetic manipulation of LC noradrenergic neurons may affect rearing by modulating MNs. To do this, we injected the Cre-dependent optogenetic activator ChrimsonR in the LC of DBH-iCre^+^ mice, a mouse line expressing the Cre recombinase specifically in LC noradrenergic neurons (Parlato et al., 2007). In the same mice, we injected the MCH promoter-dependent calcium activity indicator GCaMP6s in the LH (Fig. 5*A*). We functionally confirmed the effectiveness of optogenetic stimulation of LC DBH-ChrimsonR neurons by observing pupil dilation in response to the LC optostimulation (Fig. 5*B*; Joshi et al., 2016; Reimer et al., 2016; Privitera et al., 2020). Next, we recorded MCH-GCaMP6s cell activity in the open field, while optostimulating the LC noradrenergic neurons (Fig. 5*C*). At the onset of laser illumination, both MN activity and rearing significantly decreased in ChrimsonR-expressing mice, but not in control mice (Fig. 5*D–F*).

10.1523/JNEUROSCI.0015-24.2024.f5-1 Figure 5-1 **Effect of optostimulation and optoinhibition of LC neurons on other behaviors.** A-D) Effect of optostimulation of LC-noradrenergic neurons on behaviors. Grooming ** p = 0.0025; immobility *ns* p = 0.3933; locomotion * p = 0.0332; turning *ns* p = 0.2229; unpaired t-test; n = 6 LC-ChrimsonR expressing mice and 6 control mice. E-H) Effect of optoinhibition of LC-noradrenergic neurons on behaviors. Grooming ** p = 0.0084; immobility *ns* p = 0.1905; locomotion *ns* p = 0.5518; turning * p = 0.0205; unpaired t-test; n = 6 eNpHR-expressing mice and 5 control mice. I-L) Effect of optoinhibition of LC-noradrenergic neurons after injection of MCH-R1 antagonist SNAP on behaviors. Grooming *ns* p = 0.0980; immobility *ns* p = 0.8195; locomotion *ns* p = 0.4563; turning * p = 0.0278; unpaired t-test; n = 6 eNpHR-expressing mice and 5 control mice). Download Figure 5-1, TIF file.

10.1523/JNEUROSCI.0015-24.2024.f5-2 Figure 5-2 **Effect of optostimulation and optoinhibition of LC neurons on center/border preference and locomotor activity in the open field.** A-B) Effect of optostimulation of LC-noradrenergic neurons in control and LC-ChrimsonR mice (center/border preference * p = 0.0134; locomotor activity * p = 0.0153; unpaired t-test; n = 6 LC-ChrimsonR expressing mice and 6 control mice). C-D) Effect of optoinhibition of LC-noradrenergic neurons in control and LC-NpHR mice (center/border preference * p = 0.0202; locomotor activity *ns* p = 0.0629; n = 6 eNpHR-expressing mice and 5 control mice). E-F) Effect of optoinhibition of LC-noradrenergic neurons after injection of MCH-R1 antagonist SNAP in control and LC-NpHR mice (centre/border preference * p = 0.0432; locomotor activity *ns* p = 0.0708; n = 6 eNpHR-expressing mice and 5 control mice). Download Figure 5-2, TIF file.

10.1523/JNEUROSCI.0015-24.2024.f5-3 Figure 5-3 **Control data relating to Figures 2, 3 and 5.** A) Effect of vehicle IP injection compared to no injection (related to Fig. 4 - ***ns*** p = 0.8429, unpaired t-test, n = 15 mice injected with vehicle and 12 mice with no injection. Data are shown as mean ± SEM. ns, p > 0.05; *, p < 0.05; **, p < 0.01). B) Low-magnification image showing ChrimsonR-expressing neurons in the MCH area in the LH (related to Fig. 3). C) Assessment of conditioned freezing responses in wild-type mice utilizing a previously conditioned tone (yellow background) as an acute stressor in the open field (n = 8 mice WT mice). Download Figure 5-3, TIF file.

Next, we injected the Cre-dependent silencing opsin eNpHR into the LC and MCH promoter-driven GCaMP6s in the LH of DBH-iCre^+^ mice (Fig. 5*G*). Our pilot experiments indicated that in our standard open-field conditions, LC optosilencing did not alter MN-GCaMP signals, which could be due to a low baseline LC activity. To avoid such a “floor effect” while studying the effects of the LC optosilencing, we employed a paradigm involving an acute stressor. We first subjected mice to fear conditioning, where a tone was associated with a footshock, and then played this tone (now serving as stress-inducing cue, as shown in Extended Data Fig. 5-3C) simultaneously with optosilencing. The LC optosilencing increased MN activity (Fig. 5*H*) and increased rearing in eNpHR-expressing, but not in control, mice (Fig. 5*I–K*). The MCHR1 antagonist SNAP blocked the effect of the LC optosilencing in rearing (Fig. 5*L–N*). Note that SNAP did not further decrease rearing in control mice here, as expected from a floor effect of MCH activity under conditions of stress (González et al., 2016a). Interestingly, in addition to rearing, the LC optomodulation altered some other behaviors, like grooming and locomotion but not immobility and turning (Extended Data Fig. 5-1A–H), and these changes were largely unaffected by SNAP (Extended Data Fig. 5-1I–L). Additionally, the LC optostimulation decreased center/border preference and locomotor activity (Extended Data Fig. 5-2A,B) while LC optosilencing increased center/border preference and created a trend toward increased locomotor activity (Extended Data Fig. 5-2C,D), as expected (Valentino and Bockstaele, 2008; McCall et al., 2015; Hirschberg et al., 2017; Benarroch, 2018; Zerbi et al., 2019; Morris et al., 2020; Poe et al., 2020; Privitera et al., 2024), and the latter effect was not changed by MCHR1 antagonist SNAP (Extended Data Fig. 5-2E,F).

In summary, we found that optostimulation of LC noradrenergic neurons inhibits MN activity and rearing. On the other hand, optoinhibition of LC noradrenergic neurons disinhibits MNs and causes an increase in rearing behavior, which is abolished by treatment with the MCHR1 antagonist SNAP, indicating that the effect of LC noradrenergic neurons on rearing requires MN-derived signals. Together, these data show that, in behaving mice, LC noradrenergic neurons exert inhibitory control over MNs and MCHR1-dependent rearing behavior.

## Discussion

MNs are classically known to be involved in energy homeostasis (Qu et al., 1996; MacNeil, 2013; Petrovich, 2018; Lord et al., 2021) and sleep regulation (Konadhode et al., 2013; Ferreira et al., 2017) but have more recently been found to intervene also in learning and plasticity phenomena (Monzon et al., 1999; Varas et al., 2002, 2003; Adamantidis et al., 2005; Adamantidis and Lecea, 2009; Pachoud et al., 2010; Barillier et al., 2015; González et al., 2016a; Blanco-Centurion et al., 2019; Izawa et al., 2019; Kosse and Burdakov, 2019; Oh et al., 2019; Burdakov and Peleg-Raibstein, 2020; Concetti and Burdakov, 2021; Harris et al., 2022), exploration of objects (Blanco-Centurion et al., 2019; Kosse and Burdakov, 2019), and the stabilization of hippocampal theta rhythm (Jego et al., 2013), which is associated to spatial exploration and learning (Lopes-dos-Santos et al., 2018; Kragel et al., 2020). The activity profile of MNs has been investigated in relation to neutral stimuli (González et al., 2016a; Blanco-Centurion et al., 2019; Kosse and Burdakov, 2019), appetitive stimuli (Domingos et al., 2013; Subramanian et al., 2022), and aversive stimuli (Concetti et al., 2020). However, in exploration-like assays where MNs display phasic activation (González et al., 2016a; Blanco-Centurion et al., 2019; Kosse and Burdakov, 2019), it has not been previously tested whether MN activity spikes during acts of rearing or during other interspersed actions that occur in these assays. We now found that MN activity acutely and reversibly increases during acts of unsupported rearing—a frequently observed but relatively understudied behavior, whose neural triggers and modulators remain unclear despite its increasingly recognized relevance in both fundamental and translational neuroscience (Sutherland et al., 1982; Lever et al., 2006; Mun et al., 2015; Barth et al., 2018; Sturman et al., 2018; Chen et al., 2023; Layfield et al., 2023). Complementary pharmacological and optogenetic tools revealed MNs as a causal and specific driver of rearing behavior, controlling both its initiation and maintenance. Furthermore, we report in vivo evidence for an upstream LC→MN inhibitory signaling, which modulates rearing behavior. Together, these findings define a subcortical neural module which both tracks and controls exploratory rearing, thus defining a new element in the biology of information seeking.

Prior studies suggest that other hypothalamic neurons can exert differential control on distinct microstructural elements of self-paced behaviors (event frequency vs event duration; Johnson, 2018), such as running and eating (Karnani et al., 2020; Viskaitis et al., 2022). This did not seem to be the case for MNs and rearing, where we observed effects on both event frequency and duration (Fig. 4*A–C*), suggesting that MNs orchestrate both initiation and, possibly, maintenance of rearing in an MCHR-dependent manner (Fig. 4*D*,*E*). In our study, these effects of MNs on rearing are unlikely to be a secondary by-product of MNs’ effects on locomotion or anxiety states, since the MN manipulations that affected rearing did not affect locomotion or anxiety metrics (Figs. 2*B*,*C*, 3*C*). This is important, since some previous studies in mice have suggested that MNs may have an antilocomotive effect (Marsh et al., 2002; Segal-Lieberman et al., 2003; Zhou et al., 2005; Whiddon and Palmiter, 2013), although studies in rats do not support a role for MNs in locomotion suppression (Sanchez et al., 1997; Monzon et al., 1999; Shearman et al., 2003; Lagos et al., 2011).

Antilocomotive effects related to MCH system manipulations were not observed in the present study but we note that increased rearing can involve reduced locomotion since mice cannot cover distance effectively while on hind legs. The interpretation of chronic interventions used in these studies is complicated by compensatory effects, and more recent studies suggest that MN effects on locomotion may depend on downstream targets (Chee et al., 2019). In our acute experiments modulating MNs, mice were fully habituated to the experimental setup before testing—to avoid suppression of rearing behavior by novelty-induced stress—and locomotion parameters remained unaltered as expected (locomotor activity Figs. 3*C*, 4*D*, and immobility, locomotion and turning in Fig. 3*G–I*). The absence of differences in our study in center/border preference in the open field—a measure of anxiety-like behavior—upon experimental manipulations of the MCH system may seem in contrast with past studies reporting that blockade of the MCHR1 receptor exerts an anxiolytic effect (Borowsky et al., 2002; Roy et al., 2006; He et al., 2022). However, other studies on the role of the MCH system in anxiety-related behaviors have yielded contrasting results (Kela et al., 2003; Basso et al., 2006). Differences between our results and past studies on the involvement of MNs in anxiety-like behavior may be due to differences in experimental paradigms used, and further investigation will be needed to untangle the roles of MNs in exploration and anxiety. Finally, given that there is published evidence that MNs can release several neurotransmitters in addition to MCH neuromodulator (Jego et al., 2013; Chee et al., 2015; Schneeberger et al., 2018), it was important to determine whether the MN effects on rearing were mediated by specific transmitter(s). We found that rearing modulation evoked by optogenetic MN stimulation was abolished by SNAP (Fig. 4*D*), indicating that the SNAP-sensitive MCHR1 (the only MCH receptor in the mouse)—and thus the MCH neuropeptide—was responsible.

We identified the LC as an upstream inhibitor of MCH neurons, thus adding mechanistic insight to several observations made in the past on the reduction of rearing under stress (Lever et al., 2006; Valentino and Bockstaele, 2008; Konadhode et al., 2013; Mun et al., 2015; Benarroch, 2018; Sturman et al., 2018; Morris et al., 2020; Poe et al., 2020; Privitera et al., 2020; Osorio-Forero et al., 2022), on LC activation under stress (Valentino and Bockstaele, 2008; Benarroch, 2018; Morris et al., 2020; Poe et al., 2020), rearing suppression upon LC activation (Privitera et al., 2024), and opposite dynamics of MCH neurons and LC noradrenergic neurons (Blanco-Centurion et al., 2019; Osorio-Forero et al., 2022). Our results from experiments involving exogenous activation and inhibition of LC noradrenergic neurons suggest both MCHR-dependent and MCHR-independent streams of LC functional output. One line of evidence suggesting this is the comparison of behavioral effects of LC manipulation in the presence and absence of SNAP (Extended Data Fig. 5-2). Another is that, despite the LC→MN inhibitory link, we noted a dissociation between the effects of LC and MN interventions. While—among investigated behaviors—MN modulation specifically affected rearing (Figs. 2–4), modulation of LC noradrenergic neurons also affected other behaviors (Extended Data Fig. 5-1). Interestingly, this was paralleled by a dissociation of effects of LC noradrenergic neurons and MNs on anxiety-like behavior (Extended Data Fig. 5-2). Following experimental manipulations of the MCH system, during self-paced behaviors in a nonstressful condition, no differences were observed in anxiety-like behavior, such as center/border preference in the open field (Figs. 2*B*, 3*C*). However, when the LC was activated, increased anxiety-like behavior was observed, characterized by reduced time spent in the center and reduced total distance moved (Extended Data Fig. 5-2A,B). Conversely, inhibition of the LC significantly increased the time spent in the center (Extended Data Fig. 5-2C). Overall, this suggests that LC noradrenergic neurons exert wider behavioral effects than MNs, likely through projections to additional brain areas.

Our study complements the increasing body of knowledge uncovering the complex and integrated roles of MNs and the LH, from circuit analysis (Bittencourt et al., 1992; Elias et al., 2008; Bittencourt, 2011; Niu et al., 2012; Lima et al., 2013; Haemmerle et al., 2015; González et al., 2016a; Noble et al., 2019; Liu et al., 2022), transcriptional profiling of LH (Romanov et al., 2017; Jancsik et al., 2018; Mickelsen et al., 2019), electrophysiology (Gao and Pol, 2001; van den Pol et al., 2004; Huang and Pol, 2007; Gao, 2009; Harris et al., 2022), to behavior (Domingos et al., 2013; González et al., 2016a; Izawa et al., 2019; Kosse and Burdakov, 2019; Dilsiz et al., 2020; Subramanian et al., 2023). It also adds to studies investigating naturalistic behaviors, which are proposed to improve the translational value of rodent behavioral research (Chen et al., 2023; Shemesh and Chen, 2023). Our results also identify important directions for future work. Since the LC is known to be activated by stress (Valentino and Bockstaele, 2008; Benarroch, 2018; Morris et al., 2020; Poe et al., 2020), our findings may explain why stress reduces rearing (Sturman et al., 2018) and provides a previously unknown insight into the interplay of arousal-related LC neurons (Carter et al., 2010; Benarroch, 2018) and the learning and exploration-implicated MNs (Adamantidis and Lecea, 2009; González et al., 2016a; Izawa et al., 2019; Kosse and Burdakov, 2019; Burdakov and Peleg-Raibstein, 2020). However, to define the role of the LC→MN circuitry in stress-induced modulation of information gathering, it will need to be investigated in a wider range of contexts and stressors. In particular, the roles of other stress-related areas which are sources of inhibitory inputs to MNs, such as the amygdala and the BST (González et al., 2016a), remain to be determined. We also note that, while the LC innervates the LH (Schwarz et al., 2015), indirect (polysynaptic) effects of LC cannot be ruled out by our study and need to be further investigated. Additional experiments would also be required to understand the involvement of MNs in the multiple other proposed functions of the LC, such as network resetting, brain gain control, and the inverted U relationship between arousal and performance (Aston-Jones and Cohen, 2005; Bouret and Sara, 2005; Zerbi et al., 2019; Poe et al., 2020). Furthermore, cellular-resolution studies will be needed to assess whether rearing behavior is under control of all MNs or a specific subpopulation, to what extent that would overlap with MN activation during other awake behaviors or sleep (Blanco-Centurion et al., 2019; Izawa et al., 2019; Kosse and Burdakov, 2019; Subramanian et al., 2023), and what downstream circuits are involved. Our data reveal MNs as an appropriate genetically defined entry point for addressing these fundamental questions.
