# Supplementary figures and images for: Exploratory Rearing Is Governed by Hypothalamic Melanin-Concentrating Hormone Neurons According to Locus Ceruleus
Source: J Neurosci. 2024 Apr 4;44(21):e0015242024. doi: 10.1523/JNEUROSCI.0015-24.2024 (PMC11112542; doi:10.1523/JNEUROSCI.0015-24.2024)

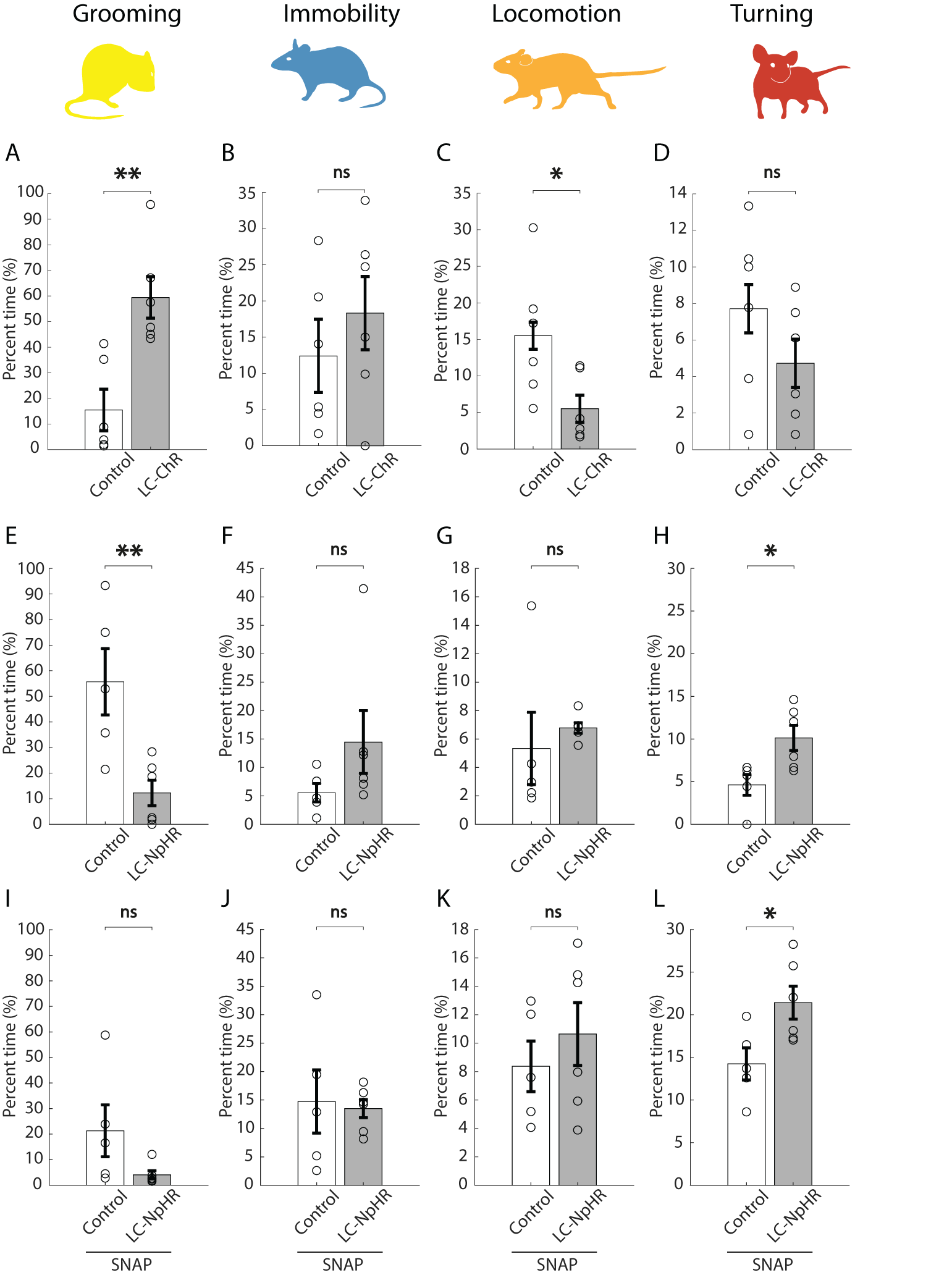

Supplement: Figure 5-1 — Effect of optostimulation and optoinhibition of LC neurons on other behaviors. A-D) Effect of optostimulation of LC-noradrenergic neurons on behaviors. Grooming ** p = 0.0025; immobility ns p = 0.3933; locomotion * p = 0.0332; turning ns p = 0.2229; unpaired t-test; n = 6 LC-ChrimsonR expressing mice and 6 control mice. E-H) Effect of optoinhibition of LC-noradrenergic neurons on behaviors. Grooming ** p = 0.0084; immobility ns p = 0.1905; locomotion ns p = 0.5518; turning * p = 0.0205; unpaired t-test; n = 6 eNpHR-expressing mice and 5 control mice. I-L) Effect of optoinhibition of LC-noradrenergic neurons after injection of MCH-R1 antagonist SNAP on behaviors. Grooming ns p = 0.0980; immobility ns p = 0.8195; locomotion ns p = 0.4563; turning * p = 0.0278; unpaired t-test; n = 6 eNpHR-expressing mice and 5 control mice). Download Figure 5-1, TIF file. [file jneuro-44-e0015242024-s001.tif]

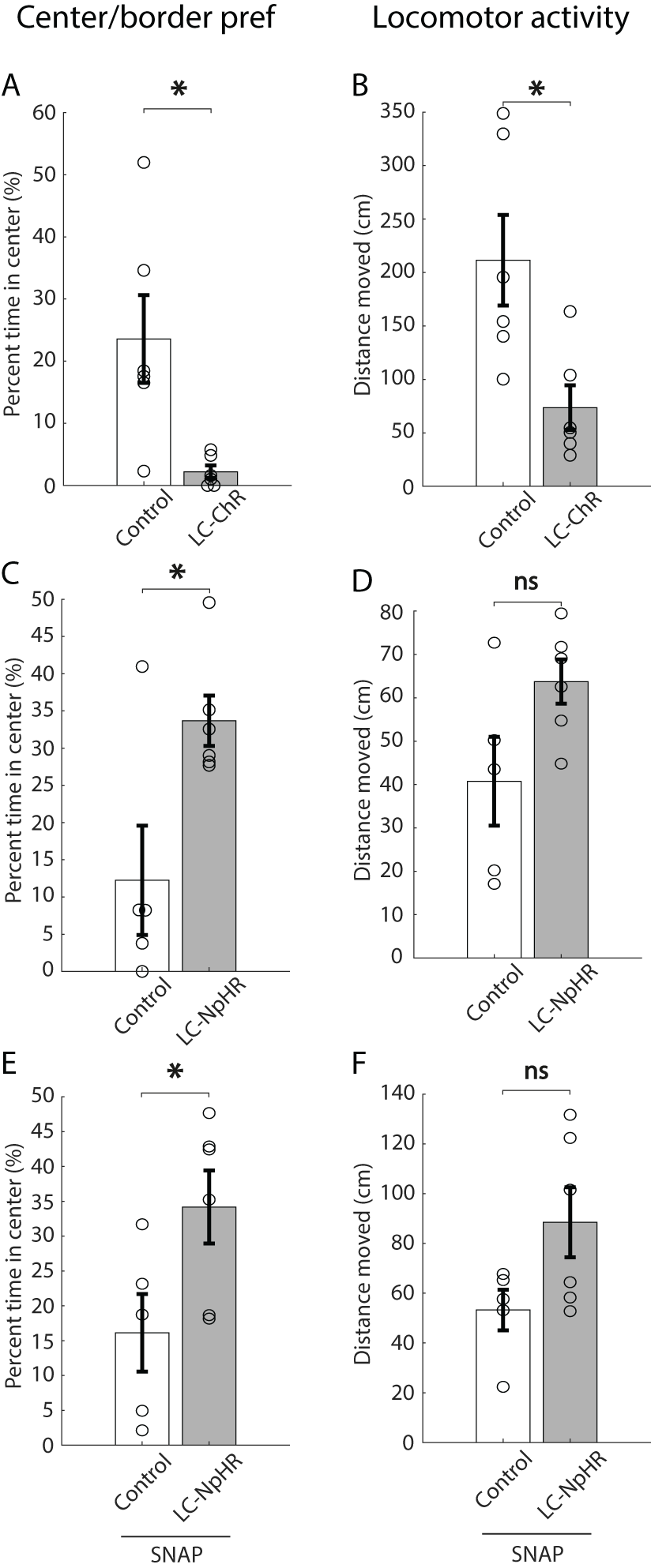

Supplement: Figure 5-2 — Effect of optostimulation and optoinhibition of LC neurons on center/border preference and locomotor activity in the open field. A-B) Effect of optostimulation of LC-noradrenergic neurons in control and LC-ChrimsonR mice (center/border preference * p = 0.0134; locomotor activity * p = 0.0153; unpaired t-test; n = 6 LC-ChrimsonR expressing mice and 6 control mice). C-D) Effect of optoinhibition of LC-noradrenergic neurons in control and LC-NpHR mice (center/border preference * p = 0.0202; locomotor activity ns p = 0.0629; n = 6 eNpHR-expressing mice and 5 control mice). E-F) Effect of optoinhibition of LC-noradrenergic neurons after injection of MCH-R1 antagonist SNAP in control and LC-NpHR mice (centre/border preference * p = 0.0432; locomotor activity ns p = 0.0708; n = 6 eNpHR-expressing mice and 5 control mice). Download Figure 5-2, TIF file. [file jneuro-44-e0015242024-s003.tif]

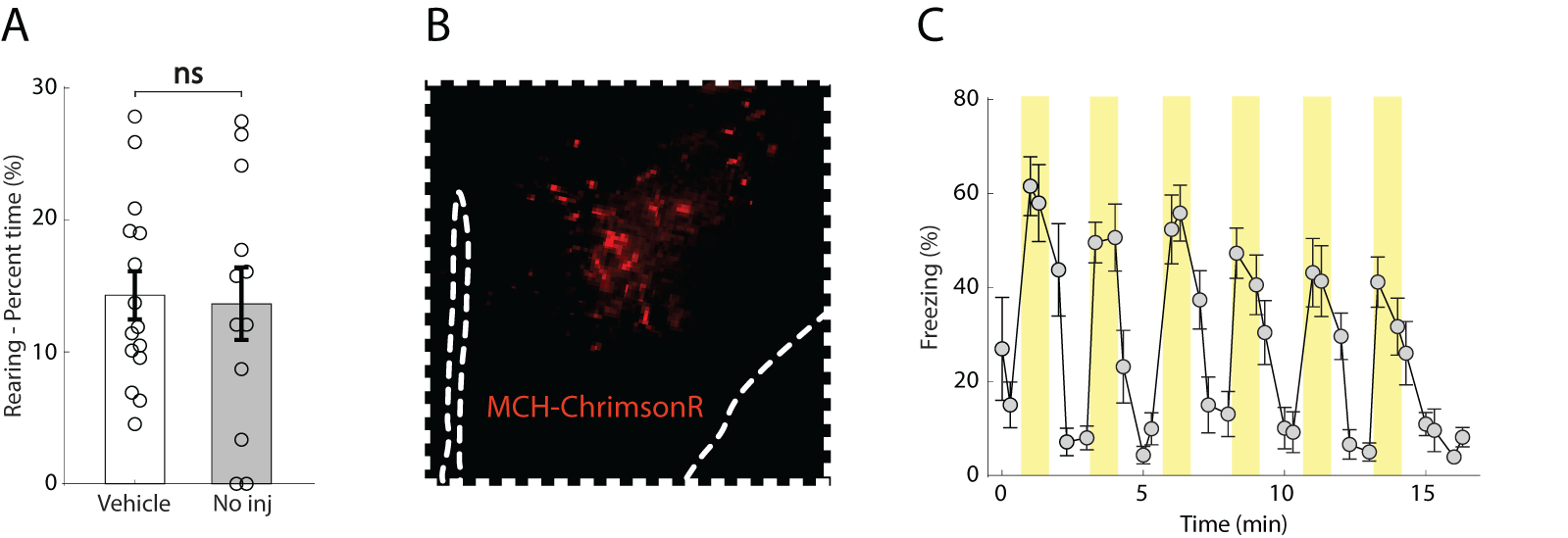

Supplement: Figure 5-3 — Control data relating to Figures 2, 3 and 5. A) Effect of vehicle IP injection compared to no injection (related to Fig. 4 - ns p = 0.8429, unpaired t-test, n = 15 mice injected with vehicle and 12 mice with no injection. Data are shown as mean ± SEM. ns, p > 0.05; *, p < 0.05; **, p < 0.01). B) Low-magnification image showing ChrimsonR-expressing neurons in the MCH area in the LH (related to Fig. 3). C) Assessment of conditioned freezing responses in wild-type mice utilizing a previously conditioned tone (yellow background) as an acute stressor in the open field (n = 8 mice WT mice). Download Figure 5-3, TIF file. [file jneuro-44-e0015242024-s002.tif]
